# *NRAMP* gene family in *Kandelia obovata*: genome-wide identification, expression analysis, and response to five different copper stress conditions

**DOI:** 10.3389/fpls.2023.1318383

**Published:** 2024-01-04

**Authors:** Quaid Hussain, Ting Ye, Chenjing Shang, Sihui Li, Asadullah Khan, Jackson Nkoh Nkoh, Abd El-Zaher M. A. Mustafa, Mohamed S. Elshikh

**Affiliations:** ^1^ Shenzhen Engineering Laboratory for Marine Algal Biotechnology, Shenzhen Public Service Platform for Collaborative Innovation of Marine Algae Industry, Guangdong Engineering Research Center for Marine Algal Biotechnology, College of Life Science and Oceanography, Shenzhen University, Shenzhen, China; ^2^ College of Physics and Optoelectronic Engineering, Shenzhen University, Shenzhen, China; ^3^ Department of Botany and Microbiology, College of Science, King Saud University, Riyadh, Saudi Arabia

**Keywords:** *Kandelia obovata*, NRAMP gene family, copper stress, phylogenetic analysis, gene expression profiling

## Abstract

Natural resistance-associated macrophage proteins (NRAMPs) are a class of metal transporters found in plants that exhibit diverse functions across different species. Transporter proteins facilitate the absorption, distribution, and sequestration of metallic elements within various plant tissues. Despite the extensive identification of *NRAMP* family genes in various species, a full analysis of these genes in tree species is still necessary. Genome-wide identification and bioinformatics analysis were performed to understand the roles of *NRAMP* genes in copper (CuCl_2_) stress in *Kandelia obovata* (Ko). In *Arachis hypogaea* L., *Populus trichocarpa*, *Vitis vinifera*, *Phaseolus vulgaris* L., *Camellia sinensis*, *Spirodela polyrhiza*, *Glycine max* L. and *Solanum lycopersicum*, a genome-wide study of the NRAMP gene family was performed earlier. The domain and 3D structural variation, phylogenetic tree, chromosomal distributions, gene structure, motif analysis, subcellular localization, cis-regulatory elements, synteny and duplication analysis, and expression profiles in leaves and CuCl_2_ were all investigated in this research. In order to comprehend the notable functions of the NRAMP gene family in *Kandelia obovata*, a comprehensive investigation was conducted at the genomic level. This study successfully found five *NRAMP* genes, encompassing one gene pair resulting from whole-genome duplication and a gene that had undergone segmental duplication. The examination of chromosomal position revealed an unequal distribution of the *KoNRAMP* genes across chromosomes 1, 2, 5, 7, and 18. The *KoNRAMPs* can be classified into three subgroups (I, II, and SLC) based on phylogeny and synteny analyses, similar to *Solanum lycopersicum*. Examining cis-regulatory elements in the promoters revealed five hormone-correlated responsive elements and four stress-related responsive elements. The genomic architecture and properties of 10 highly conserved motifs are similar among members of the NRAMP gene family. The conducted investigations demonstrated that the expression levels of all five genes exhibited alterations in response to different levels of CuCl_2_ stress. The results of this study offer crucial insights into the roles of *KoNRAMPs* in the response of *Kandelia obovata* to CuCl_2_ stress.

## 1 Introduction

Plants have developed adaptive mechanisms that facilitate the uptake of necessary nutrients and mitigate the adverse effects of heavy metal (HM) toxicity. One important process is the existence of membrane transport systems in plants, which are essential for numerous aspects of vital mineral and nutrient homeostasis as well as the accumulation, translocation, and detoxification of hazardous metals (Wu et al., 2021; Liu et al., 2022; Ma et al., 2023). Previous studies have demonstrated that NRAMPs play a crucial role as integral membrane transporters in various species, including plants. These proteins are responsible for metal uptake, transport, accumulation, and detoxification (Zhang et al., 2020; Tian et al., 2021). The *NRAMP* gene was initially identified in rats in 1993 (Vidal et al., 1993). The *NRAMPs* are a significantly conserved family of integral membrane proteins that play a crucial role in facilitating the transport of divalent metals across the cellular membrane. *NRAMP* genes have been detected in several organisms, including bacteria, fungi, plants, and mammals (Cellier et al., 1996; Segond et al., 2009). The NRAMP proteins have a high degree of conservation across various organisms, from bacteria to mammals. These proteins typically consist of 10-12 transmembrane domains (TMD) and possess a consensus transport signature (Cellier et al., 1995; Tan et al., 2023).

However, the majority of NRAMP proteins can transport several metal ions, including cobalt (Co), nickel (Ni), copper (Cu), iron (Fe), manganese (Mn), zinc (Zn), and cadmium (Cd)(Nevo and Nelson, 2006; Ishida et al., 2018). The roles of NRAMP proteins in plants have been investigated in *Arabidopsis thaliana* (Segond et al., 2009), *Camellia sinensis* (Li et al., 2021), *Spirodela polyrhiza* (Chen et al., 2021), *Glycine Max* L. (Qin et al., 2017), *Solanum tuberosum* (Tian et al., 2021), *Populus trichocarpa* (Ma et al., 2023), *Phaseolus vulgaris* L. (Ishida et al., 2018), *Arachis hypogaea* L. (Tan et al., 2023). Understanding the physiological role of these elements in plant development requires identifying a particular collection of transporters that supply the delicate balance of metal concentration across the cellular membrane (Ishida et al., 2018; Kravchenko et al., 2018). Nevertheless, the function of the NRAMP protein family and the Cu transport pathways in *Kandelia obovata* is still unclear.

Mangroves serve as a significant reservoir for HMs, with Cu, lead (Pb), and zinc being the predominant HM pollutants (Cheng et al., 2017) (Bodin et al., 2013). HMs represent a significant class of anthropogenic harmful substances within mangrove ecosystems (Huang and Wang, 2010). Among them, Cu, Zn, and Pb are often encountered pollutants (Qiu et al., 2011; Li et al., 2015). Shen et al. (2021) reported that HM enrichment on the root surface of *Kandeliaobovata* is higher than some other mangrove plants in South China, including *Acanthus ilicifolius*, *Aeagiceras corniculatum*, *Bruguiera gymnorrhiza*, and *Heritiera littorlis*. A viviparous mangrove species found in the intertidal zones of tropical and subtropical coasts, *Kandelia obovata* is a member of the Rhizophoraceae family in the Malpighiales (Sheue et al., 2003). The distribution of this species spreads from northern Vietnam to southeast China and south Japan in the East Asian region (Sheue et al., 2003; Yang et al., 2019). The native distribution of this species encompasses the Hainan, Guangdong (including Hong Kong and Macau), Guangxi, Fujian, and Taiwan Provinces in China (Li and Lee, 1997). *Kandelia obovata*, a mangrove species found in China, exhibits notable cold resistance and salt tolerance, enabling its survival in northern regions (Yang et al., 2019). Prior research has demonstrated that this species could collect HMs (Weng et al., 2012; Weng et al., 2014). *Kandeliaobovata* is a woody plant found in tidal salt wetlands in tropical and subtropical regions from East to Southeast Asia (Sheue et al., 2003; Hu et al., 2020). *Kandelia obovata* adapts to transitional ecosystems characterized by the interface between land and water by effectively coping with periodic and aperiodic tidal influences. These tidal impacts give rise to elevated salinity levels, substantial erosion, and anaerobic conditions (Giri et al., 2011; Hu et al., 2020).

Copper is a protein cofactor that participates in electron transfer activities, hence playing a crucial role in these biochemical processes. Additionally, Cu is an essential component for plants. Cu fulfills several functions in biological processes, such as photosynthesis, respiration, ethylene sensing, reactive oxygen metabolism, and cell wall remodeling (Burkhead et al., 2009). Cu is a vital plant element, but excessive quantities can lead to phytotoxicity. Soils can get contaminated by Cu and other HMs due to human activities, such as industrial, mining, and agricultural practices. These activities include sewage sludges, organic wastes, fertilizers, and fungicides. Therefore, it is crucial to investigate the correlation between HMs and plants, given their impact on crop yield and plant development (Mourato and Martins, 1900; Fageria, 2001).

As far as current information is concerned, the presence of the NRAMP gene family in *Kandelia obovata* has not been documented. Therefore, this study is the first instance in which a genome-wide analysis was conducted to identify *NRAMP* genes inside the genome of *Kandelia obovata*. This study aimed to identify and characterize five *NRAMP* genes and then investigated their expression levels in response to five CuCl_2_ treatments. In order to gain a deeper understanding of the evolution of *NRMAP* genes in *Kandelia obovata*, various aspects were examined using bioinformatics methodologies. These included the analysis of gene structures, physicochemical properties, chromosomal distribution, synteny and duplication structures, conserved motifs, cis-elements, phylogenetic relationships, subcellular localization, and the expression profiles of *NRAMP* homologs. The present study investigates the characterization and expression analysis of the *NRAMP* family in *Kandelia obovata*. This research seeks to establish a theoretical foundation for future investigations on the response of the *NRAMP* family to CuCl_2_ treatment in *Kandelia obovata* plants.

## 2 Materials and methods

### 2.1 Characterization and identification of the *NRAMP* genes in *Kandelia obovata*

For *Kandelia obovata*, the genome sequences were obtained from the NCBI database (https://www.ncbi.nlm.nih.gov/; BioProject/GWH, Accession codes: PRJCA002330/GWHACBH00000000) and the *Kandelia obovata* protein database (https://www.omicsclass.com/article/310) (Hu et al., 2020). Two databases confirm hypothetical proteins: Pfam (http://pfam.xfam.org/) and NCBI CDD (https://www.omicsclass.com/article/310, E-value 1.2 × 10^−28^). The protein sequence analysis of NRAMP linked with the domain profile was conducted utilizing the Pfam database (http://pfam.xfam.org). The *Kandelia obovata* genome database (https://www.omicsclass.com/article/310) and the National Center for Biotechnology Information (NCBI) database (https://www.ncbi.nlm.nih.gov/) were employed to identify and validate five NRAMP family genes, namely *KoNRAMP1*, *KoNRAMP2*, *KoNRAMP3*, *KoNRAMP4*, and *KoNRAMP5*. Protparam (http://web.expasy.org/protparam/) was utilized to obtain physicochemical properties (Si et al., 2023).

### 2.2 Chromosomal distribution

Using the NCBI database and https://www.omicsclass.com/article/310, the genomic locations and protein sequences of every *NRAMP* gene in *Kandelia obovata* were identified. The distribution positions of *NRAMP* genes on chromosomes were also evaluated. The MapGene2Chromosome (MG2C) tool was utilized to determine the chromosomal locations of *NRAMP* genes in *Kandelia obovata*. The MG2C tool, version 2.0, was used at the URL: http://mg2c.iask.in/mg2c (Hussain et al., 2022a).

### 2.3 Phylogenetic tree construction

Protein sequences of *NRAMP* genes from the following species were used in the phylogenetic analysis: *Vitis vinifera* (Vv), *Aegiceras corniculatum* (Ac), *Solanum tuberosum* (St), *Populus trichocarpa* (Pt), *Kandelia obovata* (Ko), and *Arabidopsis thaliana* (At). The MEGA11 (V 6.06) software, available at www.megasoftware.net, was commonly employed to align protein sequences (Hashemipetroudi et al., 2023). The phylogenetic tree was constructed using the neighbor-joining (NJ) method, using 1000 bootstrap replicates. Using Fig Tree V1.4.4, the phylogenetic tree was examined and modified (Hussain et al., 2023).

### 2.4 Gene structure and significant motif analyses

The genome of *Kandelia obovata* has been revealed to include five genes belonging to the NRAMP family. The exon/intron arrangements of the five *NRAMP* genes were exhibited along with the structural analyses of the genes via web software (http://gsds.cbi.pku.edu.cn) (Shang et al., 2023). More conserved strings or sections were found in the protein sequences of the five NRAMP proteins, according to the online tool MEME v5.4.1, which can be accessed at https://meme-suite.org/meme/tools/glam2scan (Shang et al., 2023). The application employed the following settings: sequencing of alphabet DNA, RNA, or protein; site distribution limited to zero or one occurrence per sequence (zoops); motif finding mode set to classic mode; and 10 motifs. Using the TBtools program, the MEME findings were displayed following downloading the relevant mast file (Hussain et al., 2022b).

### 2.5 Synteny and duplication analysis

The Minspan (Available online) generated synteny associations of *NRAMP* genes from the following species: *Vitis vinifera* (Vv), *Aegiceras corniculatum* (Ac), *Solanum tuberosum* (St), *Populus trichocarpa* (Pt), and *Kandelia obovata* (Ko). With the KaKs Calculator 2.0 (https://sourceforge.net/projects/kakscalculator2/), the synonymous (Ks), non-synonymous (Ka), and Ka/Ks ratios were computed in order to examine the evolutionary constraints of each pair of *NRAMP* genes (Hussain et al., 2023).

### 2.6 Cis-regulatory elements

The NRAMP family members’ 2,500 bp upstream sequences were gathered using the *Kandelia obovata* genome assembly database. The PlantCARE tool (http://bioinformatics.psb.ugent.be/webtools/plantcare/html/) was employed to identify CREs from the obtained sequences. TBtools was used to generate the most prevalentCREs identified for the *NRAMP* genes, following a comprehensive analysis of the frequency of each CRE motif (Yang et al., 2022; Si et al., 2023; Yaghobi and Heidari, 2023).

### 2.7 3D structure and subcellular localization

The three-dimensional (3D) structure can be estimated using SWISS-MODEL (https://swissmodel.expasy.org/interactive) (Shang et al., 2023). The subcellular localization of the NRAMP family genes was predicted using two online tools.

ProtComp 9.0, available at http://linux1.softberry.com/berry.phtml?topic=protcomppl&group=programs&subgroup=proloc, is a software tool used (Si et al., 2023).

The CELLO server, which can be visited at cello.life.nctu.edu.tw (Hussain et al., 2023; Sajjad et al., 2023).

### 2.8 Plant material and environmental conditions

In the experiments, one-year-old *Kandelia obovata* seedlings were planted in the mangrove conservation site (109.22° N, 21.42° E) of Golden Bay Mangrove Reserve in Beihai, Guangxi Province. The soil was irrigated with CuCl_2_ irrigation every six months and was watered with the nearby seawater every morning and evening as part of semi-natural cultivation techniques. Five different concentrations of CuCl_2_—0, 50, 100, 200, and 400 mg/L—were utilized for the Cu0, Cu50, Cu100, Cu200, and Cu400 treatments, respectively, throughout two years. Cu0 mg/L was used as the starting concentration for the control treatment, which only used local seawater. The soil sample used for this experiment had a background Cu concentration of < 1.0%, which was classified as non-polluted (Liao et al., 2023). After two years of treatment, plant samples were gathered to evaluate the parameters (Hussain et al., 2023).

### 2.9 Quantitative real-time PCR assays

Total RNA extraction from the leaves was performed using TRIzol (Invitrogen, http://www.invitrogen.com). The ABI PRISM 7500 Real-time PCR Systems, manufactured by Applied Biosystems, were utilized in this study to perform quantitative real-time PCR (qRT-PCR) assays. The 2^−∆∆CT^ approach, as previously outlined (Hussain et al., 2022c; Sun et al., 2022), was employed for data analysis. The actin gene of *Kandelia obovata* (KoActin) was used as a reference gene using the sequence given by Sun et al., 2022 (forward primer: CAATGCAGCAGTTGAAGGAA, reverse primer: CTGCTGGAAGGAACCAAGAG). The *KoNRAMPs* gene primers utilized in real-time PCR are listed in **Table 1** and were created with the PRIMER 5.0 software (http://www.premierbiosoft.com) (Hussain et al., 2023).

**Table 1:** The primers utilized in this study’s qRT-PCR gene expression investigation.

Gene name	Primer name	Sequence (5’ to 3’)
*KoNRAMP1*	Forward	AATTGATCGTGTGGCCGGAA
	Reverse	CCAGACACACTCTTCCCCAC
*KoNRAMP2*	Forward	GCATGGATGGTGGACAGTGA
	Reverse	TTTCTCGCAACTCCCCGTAG
*KoNRAMP3*	Forward	AGACTCAAACCCTGTGGCTG
	Reverse	CTGGTCGGCTTTCCATGAGT
*KoNRAMP4*	Forward	CTCCCAATCCCGACAAGAGC
	Reverse	TTCATCTGTGTCCCCTCCCT
*KoNRAMP5*	Forward	GGGAGCCAGCAACCTAATGA
	Reverse	GGTCCGGCTACAAGCACTAA

### 2.10 Statistical analysis

The data was analyzed using Statistix 8.1, an analytical software developed in Tallahassee, FL, USA. A one-way ANOVA was employed to evaluate the data, and the results were presented as the mean and standard deviation (SD) of the three replicates. Five distinct Cu stress plants (Cu0, Cu50, Cu100, Cu200, and Cu400 mg/L) were compared for differences in leaf mean values using an LSD (least significant difference) test at p < 0.05 (Khan et al., 2022). The statistical tool GraphPad Prism version 9.0.0 for Windows, developed by GraphPad Software, located in San Diego, California, USA (https://www.graphpad.com), was used to construct the graphs (Asim et al., 2022).

## 3 Results

### 3.1 Identification of NRAMP family members in *Kandelia obovata*

**Figure 1:**
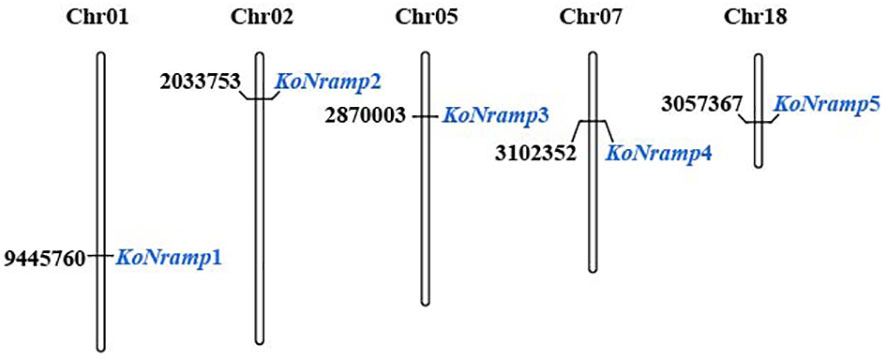
The schematic representation displays the distribution of the *NRAMP* gene on the five chromosomes of *Kandelia obovata*. The gene’s name is indicated in blue on the right side. The chromosomal regions where the *NRAMP* genes are located are denoted by black letters. The chromosomal numbers can be observed at the uppermost region of each chromosome. The chromosomal numbers can be found at the uppermost region of each chromosome (Chr).

The total number of *NRAMP* genes found in the genome of *Kandelia obovata* is five, which is significantly more than the number of *NRAMPs* found in other plant species such as *Arabidopsis thaliana*, peanut (*Arachis hypogaea* L.), common bean (*Phaseolus vulgaris*), potato (*Solanum tuberosum*), soybean (*Glycine Max* L.), *Spirodela polyrhiza*, and tea plant (*Camellia sinensis*). The molecular weight of the NRAMP family exhibited ranging from 49.4 to 139, with an average value of 84.12 kilodaltons (kDa). The protein KoNRAMP5 exhibited the highest isoelectric point (pI) of 7.61, while *KoNRAMP* displayed the lowest pI of 5.01. The isoelectric point (pI) of the NRAMP family exhibited an average range of 5.01 to 7.61. The hydrophobic nature of five *NRAMPs* was demonstrated by the range of their grand average hydropathy index (GRAVY) values, which varied from -0.082 to 0.608. Identifying the subcellular localization of NRAMP proteins can enhance the accessibility of comprehending their molecular activity. Five *NRAMPs* were probably located in the plasma membrane, based on the subcellular localization prediction of NRAMP proteins (**Table 2**). The NRAMP family exhibited an average amino acid length of 452 to 1272, an aliphatic index of 89.87 to 120.7, and an instability range of 30.07 to 48.94. The MapChart web tool was utilized to map the genomic chromosomal distribution of the discovered *NRAMP* genes in *Kandelia obovata* by utilizing their respective chromosomal positions and assigning them to the corresponding chromosomes. The chromosomal locations of the five *NRAMP* genes, namely *KoNRAMP1*, *KoNRAMP2*, *KoNRAMP3*, *KoNRAMP4*, and *KoNRAMP5*, were identified as chromosome 01 (Chr01), chromosome 02 (Chr02), chromosome 05 (Chr05), chromosome 07 (Chr07), and chromosome 18 (Chr18) (**Figure 1**; **Table 2**).

**Table 2:** Comprehensive details of the NRAMP gene family found in *Kandelia obovata*.

Name	Gene ID	Location	AA^1^	Chains^2^	MW^3^/kDd	pI^4^	GRAVY^5^	Aliphatic Index	Instability	Subcellular localization
KoNRAMP1	geneMaker00017339	Chr1 9445760-9454057	1272	+	139	5.49	0.022	94.54	42	Plasma membrane
KoNRAMP2	geneMaker00008219	Chr2 2033753-2037135	516	+	56.6	5.01	0.608	117.8	30.7	Plasma membrane
KoNRAMP3	geneMaker00005552	Chr5 2870003-2878317	1097	–	119	6.11	-0.082	89.87	48.94	Plasma membrane
KoNRAMP4	geneMaker00003484	Chr7 3102352-3106139	452	–	49.4	5.28	0.457	113.1	40.59	Plasma membrane
KoNRAMP5	geneMaker00018842	Chr18 3057367-3065595	523	–	56.6	7.61	0.602	120.7	30.07	Plasma membrane

### 3.2 Variation across the NRAMP family in terms of domain and 3D structure

**Figure 2:**
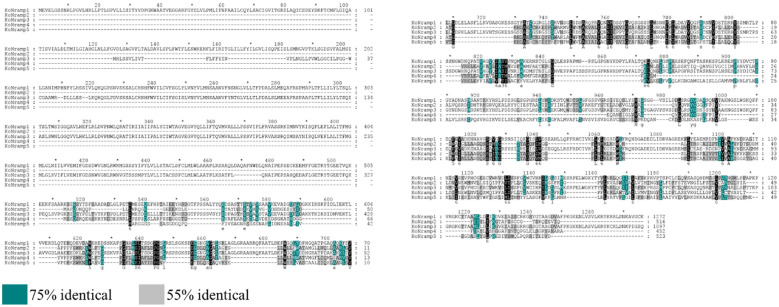
Multiple alignments of the amino acid sequence were performed utilizing information from each *KoNRAMP* transporter. The predicted transmembrane regions are displayed as black boxes. The symbols “*” that appear above the sequence indicate every 10 residues of amino acids.

**Figure 3:**
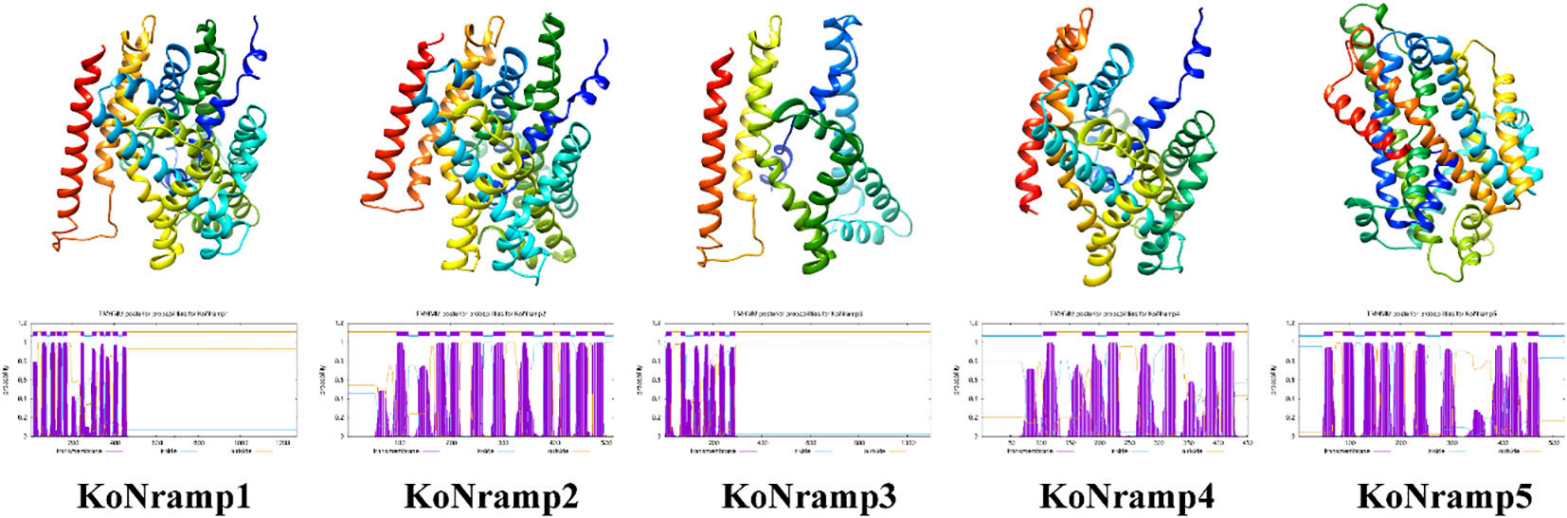
The 3D and transmembrane structures of *KoNRAMP*. The SWISS-MODEL software is utilized for the prediction of three-dimensional structural homology models. The SOSUI program validated the transmembrane structures.

Aligning all *KoNRAMPs*, BLAST in NCBI revealed that their amino acid sequences are 55–75% similar (**Figure 2**). *NRAMPs* were discovered in three transmembrane domains (TMD) of *Kandelia obovata*, according to domain analysis (PF01566) (**Figure 2**). The protein structures of *KoNRAMPs* were confirmed using the SWISS-MODEL workspace and SOSUI tool, as depicted in **Figure 3**. The significant conservation seen in all investigated species suggests that the crystal structure of a divalent metal transporter. The NRAMP proteins of *Kandelia obovata* exhibit secondary structures that are analogous to NRAMP proteins seen in other species, as depicted in **Figure 3**. These proteins also demonstrate a sequence identity and similarity ranging from 55% to 75%.

### 3.3 NRAMP protein phylogenetic relationships

**Figure 4:**
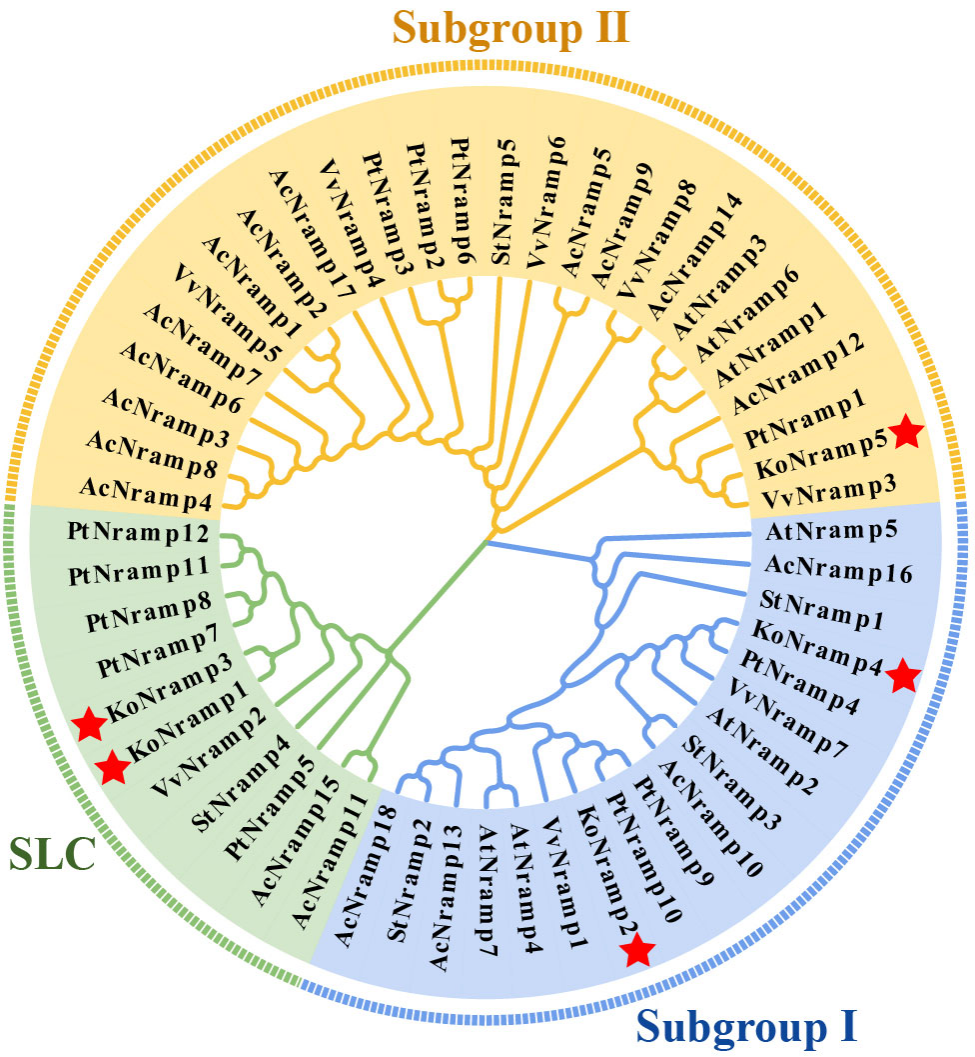
The most significant likelihood technique was employed to conduct a phylogenetic analysis of NRAMP proteins derived from *Kandelia obovata* (Ko), *Arabidopsis thaliana* (At), *Solanum tuberosum* (St), *Populus trichocarpa* (Pt), *Vitis vinifera* (Vv), and *Aegiceras corniculatum* (Ac). Subgroup I, Subgroup II, and SLC are the three groups of NRAMP proteins; a distinct color denotes each. The red asterisk symbol (*) was used to represent the *Kandelia obovata* NRAMP proteins.

An unrooted NJ tree was constructed by aligning five KoNRAMP, six AtNRAMP, five StNRAMP, twelve PtNRAMP, eight VvNRAMP, and eight AcNRAMP from *Kandelia obovata* (Ko), *Arabidopsis thaliana* (At), *Solanum tuberosum* (St), *Populus trichocarpa* (Pt), *Vitis vinifera* (Vv), and *Aegiceras corniculatum* (Ac). This allowed for the phylogenetic relationships among the NRAMP proteins from these five species. Three subgroups (I, II, and SLC) of NRAMP proteins could be identified based on the phylogenetic tree: Subgroup I consisted of 18 NRAMP proteins, which were classified into several species-specific groups. Specifically, this subgroup comprised two KoNRAMP proteins (KoNRAMP2/4), four AtNRAMP proteins (AtNRAMP2/4/5/7), three StNRAMP proteins (StNRAMP1/2/3), three PtNRAMP proteins (PtNRAMP4/9/10), two VvNRAMP proteins (VvNRAMP1/7), and four AcNRAMP proteins (AcNRAMP10/13/16/18). Subgroup II comprised of a total of 26 NRAMP proteins, including one KoNRAMP protein (KoNRAMP5), three AtNRAMP proteins (AtNRAMP1/3/6), one StNRAMP protein (StNRAMP5), four PtNRAMP proteins (PtNRAMP1/2/3/6), five VvNRAMP proteins (VvNRAMP3/4/5/6/8), and 12 AcNRAMP proteins (AcNRAMP1/2/3/4/5/6/7/8/9/12/14/17). The subgroup SLC comprised a total of eleven NRAMP proteins, including two KoNRAMP proteins (KoNRAMP1/3), one StNRAMP protein (StNRAMP4), five PtNRAMP proteins (PtNRAMP5/7/8/11/12), one VvNRAMP protein (VvNRAMP2), and two AcNRAMP proteins (AcNRAMP11/15). Hence, it can be observed from **Figure 4** that Subgroup I and SLC had a higher number of NRAMP members compared to Subgroup II.

### 3.4 *NRAMP* genes structure and conserved motifs investigation

**Figure 5:**
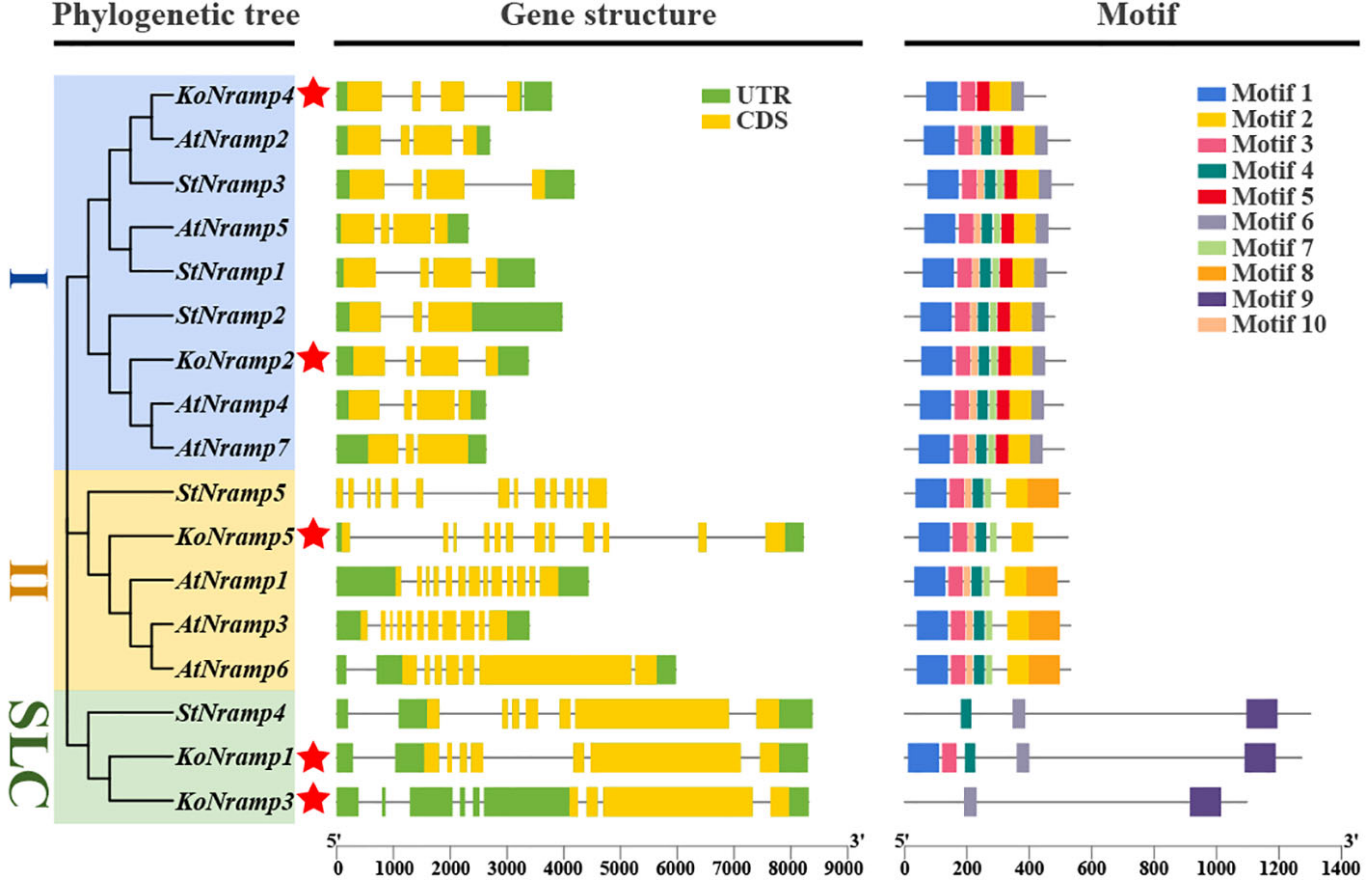
*Kandelia obovata’s NRAMP* family genes were examined for their gene structure and motif makeup. The *NRAMP* genes of both genomes were classified into two distinct categories according to their evolutionary ties—the gene structure of the *NRAMPs*. The color green visually represents the UTR regions, while the CDS or exons are depicted in yellow. A black horizontal line indicates the presence of introns. Additionally, the conserved motif structures identified in the *NRAMPs* are denoted by the letter (Ko). Various colored boxes display distinct motifs. The red asterisk symbol (*) was used to represent the *Kandelia obovata* NRAMP proteins.

A phylogenetic tree was constructed utilizing the individual sequences of the NRAMP protein. The NRAMP proteins were categorized into three categories: I, II, and SLC. This classification scheme aligns with the subsequent evolutionary groups that are elaborated upon. The homologous gene pairs exhibiting significant sequence similarities demonstrated strong evolutionary connections and similar exon-intron organizations, as illustrated in **Figure 5**. In order to further our understanding of the evolutionary dynamics of the NRAMP gene family, an investigation was conducted on the exon-intron arrangements of the *NRAMP* genes. Subgroup I consists of two constituents from the *KoNRAMPs* group, accounting for two out of four members. The presence of four exons characterizes each constituent within this subgroup. It is worth noting that the *KoNRAMP* constituent comprises four introns, while the *KoNRAMP2* constituent contains three introns.

Similarly, just one *KoNRAMP5* gene with 13–14 exons and 12–13 introns is present in subgroup II. Within the subgroup SLC, it has been observed that most *NRAMP* genes had an elongated coding sequence (CDS), accompanied by five comparatively shorter CDSs within the SLC subtribe. Furthermore, these genes have a remarkably conserved structure. Most genes in each of the three categories contained a surprising number of CDSs and introns, suggesting their activities might be very similar.

The protein sequences of *NRAMPs* were analyzed, identifying 10 conserved motifs (**Figure 5**). The conserved motifs seen in all *NRAMP* genes exhibited a range of two to eight; similarly, the *KoNRAMP* genes displayed motifs ranging from two to eight. The KoNRAMP3 protein has two distinct motifs at positions 6 and 9, whereas KoNRAMP1 and KoNRAMP4 proteins possess five motifs. The protein KoNRAMP5 exhibited a total of six distinct motifs, whereas KoNRAMP2 displayed a larger repertoire with a total of eight motifs. Most KoNRAMP proteins are anticipated to possess motifs one and three, except for KoNRAMP3.

Conversely, motif 4 has been detected in KoNRAMP1/2/5 proteins. Motifs 1 and 3 were identified in all genes except for KoNRAMP3 and StNramp4, while motif 2 was identified in all genes except KoNRAMP1/3 and StNRAMP4. Similarly, motif 4 was observed in all genes except for KoNRAMP3/4.

### 3.5 Cis-regulatory elements in the promoters of five *NRAMP* genes

**Figure 6:**
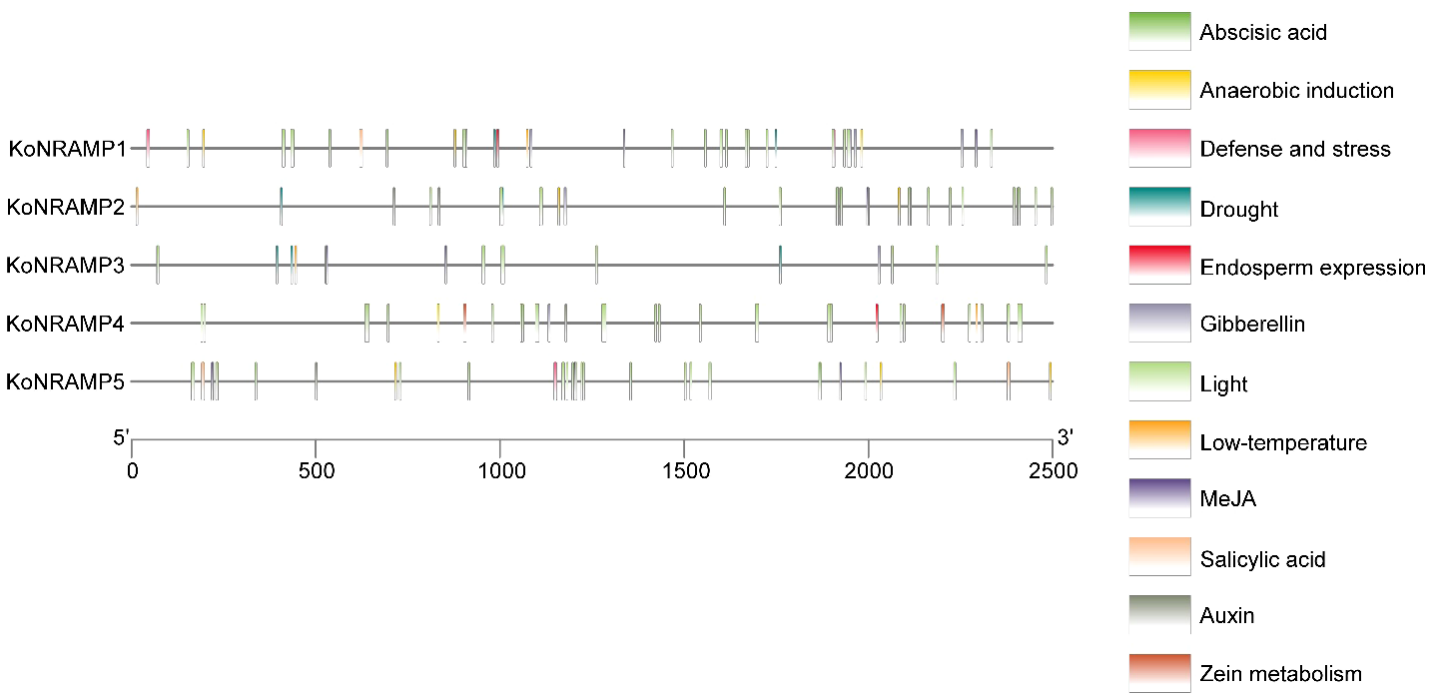
The promoters of the *KoNRAMP* gene have been found to include regulatory elements referred to as CREs. The positional distribution of the predicted CREs on the *KoNRAMP* promoters is shown by vertical bars. The promoter sequences (2500 bp) of five *KoNRAMP* genes were analyzed using PlantCARE. In the context of this legend, distinct cis-elements were symbolically represented by various colors.

After looking at the cis-element research, which may provide insights into regulatory gene expression pathways, we looked at the 2500-bp upstream promoter sequences of *NRAMP* genes. Concerning the *NRAMP* genes, it is noteworthy that the light-responsiveness gene exhibits the greatest abundance of cis-elements, with a total of 91. Furthermore, the promoter sequences of *NRAMP* genes were found to contain 53 cis-elements associated with phytohormones, including Abscisic acid (18), Gibberellin (8), Auxin (8), Salicylic acid (3), and MeJA (16). Additionally, three cis-elements related to defense and stress responses, namely low temperature (4), drought (7), and Endosperm expression (2), as well as two cis-elements associated with Zein metabolism (2), one cis-element linked to Cell cycle (1), and nine cis-elements involved in Anaerobic responses were also identified (**Figure 6**). The observed variability in the response components provides evidence for the regulatory roles of *NRAMP* genes in a wide range of physiological and biological processes.

### 3.6 Synteny and duplication analysis of NRAMP gene family

**Figure 7:**
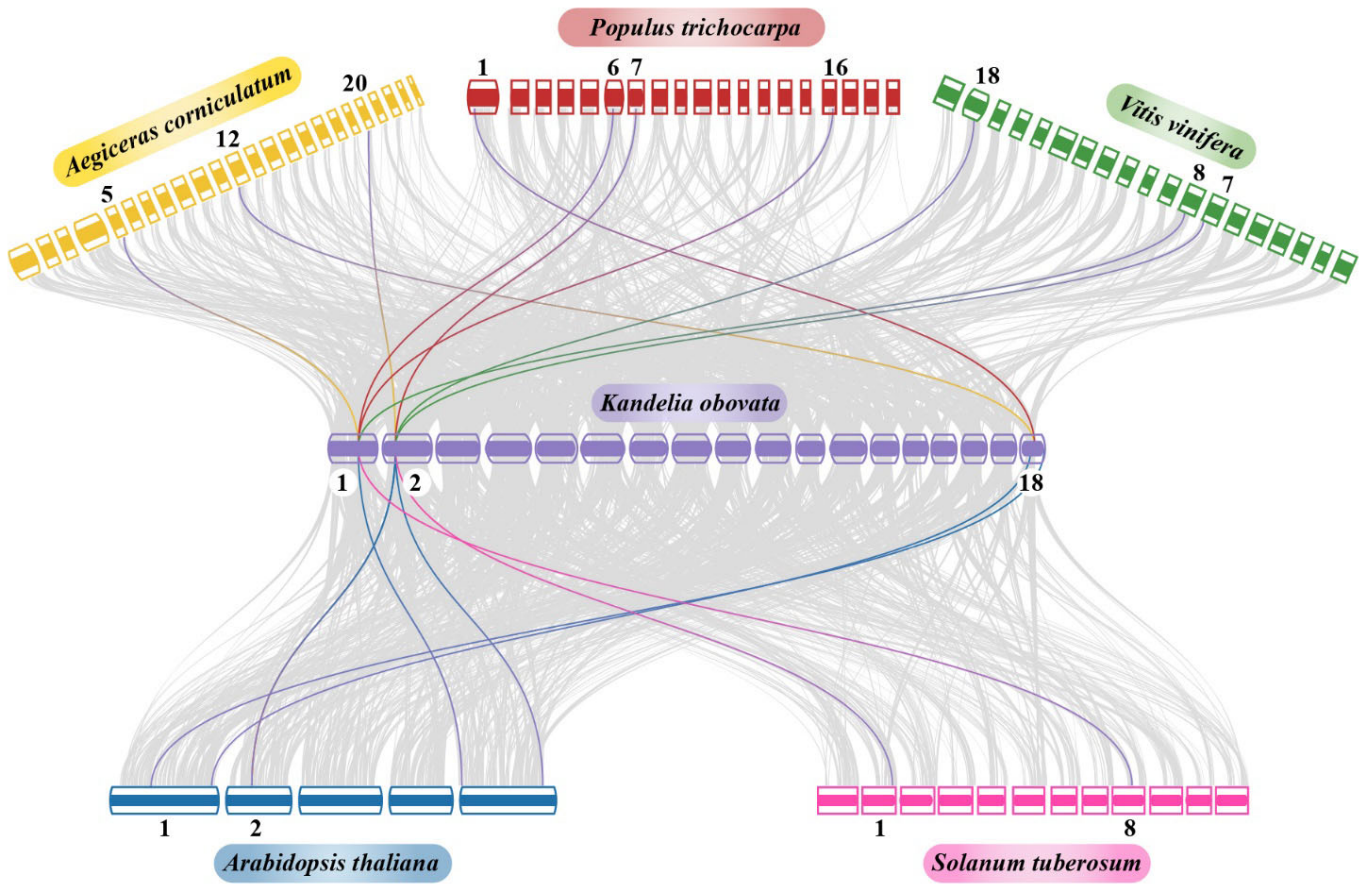
Synteny analysis of *NRAMP* genes in the chromosomes of *Aegiceras corniculatum*, *Vitis vinifera*, *Populus trichocarpa*, *Solanum tuberosum*, *Arabidopsis thaliana*, and *Kandelia obovata* was examined. The grey lines in the background show the syntenic NRAMP gene pairs, and the collinear blocks in the genomes of *Kandelia obovata* and the other five plant species are highlighted by the red lines. The box’s distinct colors represented plant species. The chromosomal number of each species is indicated at the upper and lower ends of each chromosome.

A collinearity analysis revealed significant orthologs of the *NRAMP* genes in *Kandelia obovata* and the other six inherited plant species: *Arabidopsis thaliana*, *Solanum tuberosum*, *Populus trichocarpa*, *Vitis vinifera*, and *Aegiceras corniculatum* (**Figure 7**). In chromosome 01, a gene from *Kandelia obovata* had syntenic associations with two genes from *Populus trichocarpa*, as well as with one gene each from *Arabidopsis thaliana*, *Solanum tuberosum*, *Vitis vinifera*, and *Aegiceras corniculatum*. In contrast, it is seen that within chromosome 02, a specific gene of *Kandelia obovata* exhibits syntenic relationships with two genes of *Arabidopsis thaliana* and *Vitis vinifera*. This gene demonstrates a syntenic link with one gene each from *Solanum tuberosum*, *Populus trichocarpa*, and *Aegiceras corniculatum*.

Similar syntenic relationships were seen in chromosome 18, where one gene from *Kandelia obovata* showed up with two genes from *Arabidopsis thaliana*, one gene from *Populus trichocarpa*, and one gene from *Aegiceras corniculatum* (**Figure 7**). It is worth mentioning that multiple homologs of *Kandelia obovata* (referred to as *KoNRAMPs*) have demonstrated their ability to persistently coexist with *Arabidopsis thaliana*, *Solanum tuberosum*, *Populus trichocarpa*, *Vitis vinifera*, and *Aegiceras corniculatum* through a syntenic association. This finding suggests that both segmental repetition and whole-genome duplication played significant roles in the evolutionary development of the KoNRAMPs gene family.

**Figure 8:**
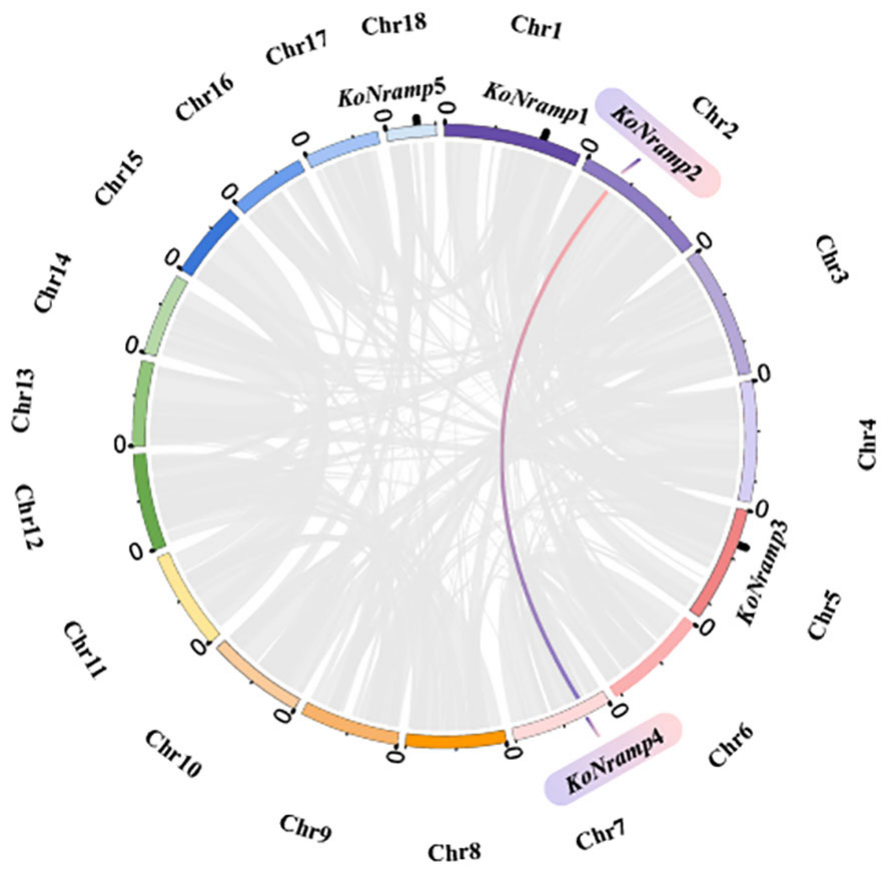
Circles represent the distribution of the *KoNRAMP* gene chromosomes and the interactions between chromosomes. The red and blue lines represent the syntenic NRAMP gene pair, whereas the syntenic blocks in the *Kandelia obovata* genome are depicted by the grey lines in the background.

Segmental and tandem duplication promotes the emergence of new gene families and plant genomes. In order to gain a deeper comprehension of the duplication events of the *Kandelia obovata NRAMP* gene, an examination was conducted on both segmental and tandem duplications within the KoNRAMP gene family. The chromosomal dispersals of five *KoNRAMP* genes were evaluated. The findings of this study indicate that a single segmental duplication event involving the *KoNRAMP2* and *KoNRAMP4* gene pairs of chromosomes A02 and A07 was observed, as depicted in **Figure 8**. It is worth noting that the *KoNRAMP* gene was not present in the remaining chromosomes. Tandem repeats paralogous genes were not identified in regions A01, A05, and A18, each with a solitary gene irrespective of the chromosome. The results of this study have illustrated the importance of duplication events in the expansion of the *KoNRAMP* family genes.

In order to further the understanding of the evolutionary constraints on the KoNRAMP gene family, an analysis was conducted to determine the values of Ka (non-synonymous substitution rate), Ks (synonymous substitution rate), and the Ka/Ks ratio for *Kandelia obovata*. The duplicated *KoNRAMP* gene pairs displayed a Ka/Ks ratio of 0.86, indicating that the *NRAMP* family genes in *Kandelia obovata* may have experienced selection pressure or a discriminatory load during their evolutionary development (**Table 3**).

**Table 3:** Comprehensive data regarding the Ka, Ks, and Ka/Ks ratio in *Kandelia obovata*.

Name	Method	*Ka*	*Ks*	*Ka/Ks*	Divergence-time (MYB)	Duplicated type
*KoNRAMP2*. *KoNRAMP4*	MS	0.06	0.07	0.86	2.2	Segmental

### 3.7 Expression analysis of *KoNRAMP* genes under CuCl_2_ treatment

**Figure 9:**
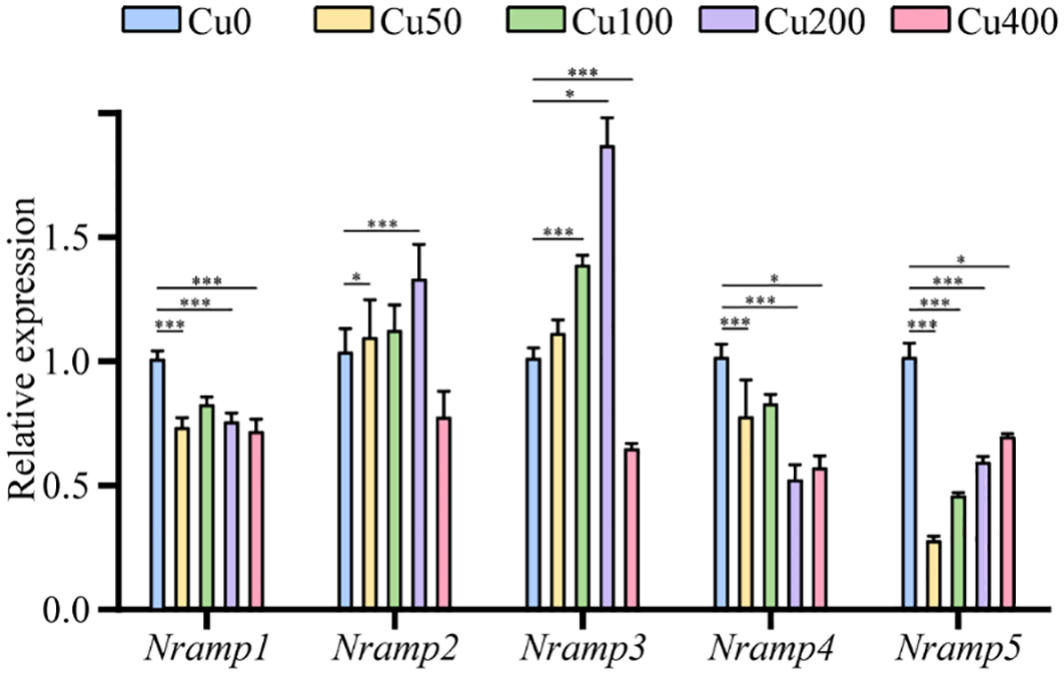
Expression of *KoNRAMPs* in leaves of seedling-stage *Kandelia obovata* plants under different Cu stress conditions (Cu0, Cu50, Cu100, Cu200, and Cu400 mg/L) as determined by qRT-PCR analysis. There is a statistically significant difference (p < 0.05) seen between the control group and all experimental conditions, as determined by the Least Significant Difference (LSD) test. *p < 0.05, *** means p < 0.001. The vertical axis shows the relative gene expression, and the horizontal axis shows *NRAMP1–5* genes.

The expression profiling of five *KoNRAMP* genes (*KoNRAMP1*, *KoNRAMP2*, *KoNRAMP3*, *KoNRAMP4*, and *KoNRAMP5*) was conducted using qRT-PCR. This analysis was carried out in the presence of five distinct CuCl_2_ treatments, namely Cu0, Cu50, Cu100, Cu200, and Cu400 mg/L, as depicted in **Figure 9**. In the present investigation, the expression levels of *KoNRAMP1*, *KoNRAMP4*, and *KoNRAMP5* were significantly reduced across all five CuCl_2_ treatments compared to the control group (Cu0). The expression levels of all five genes were shown to be significantly down-regulated in the Cu400 condition compared to the Cu0 condition. This observation can be attributed to the increased *KoNRAMPs* expression levels in the leaf when Cu availability was limited. However, when Cu was abundant (Cu400), the transcript levels of these genes decreased in comparison to both the Cu0 condition and other levels of Cu. The expression levels of *KoNRAMP2* and *KoNRAMP3* genes did not show statistical significance when comparing Cu50 with the control group. However, these genes exhibited statistically significant up-regulation in the Cu100 and Cu200 groups compared to the Cu0 group.

## 4 Discussion

Numerous *NRAMP* genes have been found and functionally described as a result of their significant role in the absorption and transport of metal ions (Thomine et al., 2003; Cailliatte et al., 2010; Sasaki et al., 2012; Peris-Peris et al., 2017; Chang et al., 2020). However, *Kandelia obovata* does not have comprehensive information about this family. The genome of *Kandelia obovata* was used in this investigation to identify five *NRAMP* genes that are relevant to reported plant species, including *Arabidopsis thaliana* (six) (Segond et al., 2009), *Camellia sinensis* (11) (Li et al., 2021), *Spirodela polyrhiza* (three) (Chen et al., 2021), *Glycine Max* L. (13) (Qin et al., 2017), *Solanum tuberosum* (five) (Tian et al., 2021), *Populus trichocarpa* (11) (Ma et al., 2023), *Phaseolus vulgaris* L. (seven) (Ishida et al., 2018), *Arachis hypogaea* L. (15) (Tan et al., 2023). Based on the findings of previously published investigations, it has been observed that numerous plant species possess a range of three to fifteen *NRAMP* genes. Our research outcome aligns with a prior investigation in this regard.

Fifteen (15) *NRAMP* family genes were found in the genome of *Arachis hypogaea* and subsequently designated as *AhNRAMP1-AhNRAMP15*. The upregulation of the majority of *AhNRAMPs* is triggered by iron deficiency in the roots of *Arachis hypogaea*, and there is a positive correlation between their expression and the accumulation of cadmium. This suggests that *AhNRAMPs* play a significant role in the interactions between iron and cadmium in *Arachis hypogaea* (Tan et al., 2023). Thirteen (13) *NRAMP* family genes have been discovered from *Glycine Max* L. Among these genes, *GmNRAMP1-13* exhibit distinct regulatory patterns in response to shortages in nitrogen, phosphorus, potassium, Fe, and sulfur, as well as toxicities caused by excessive amounts of Fe, Cu, Cd, and Mn. According to the results of the expression study, it has been indicated that *GmNRAMP* genes exhibit functionality across many tissues and developmental stages (Qin et al., 2017).

A comprehensive set of 11 *NRAMP* family genes was discovered in *Populus trichocarpa* and *Camellia sinensis* genome*s*. These genes were subsequently designated as *PtNRAMP1-PtNRAMP11* and *CsNRAMP1-CsNRAMP11*. According to the findings of gene expression research, it was observed that the *PtNRAMP* genes exhibited varying responses to metal stress conditions, such as deficiencies in Fe and Mn, as well as toxicities caused by Fe, Mn, Zn, and Cd (Ma et al., 2023). To evaluate the effects of Pb treatment on roots and leaves, the expression levels of *CsNRAMPs* were found (Li et al., 2021). A comprehensive analysis has shown the identification of *NRAMP* family genes in *Phaseolus vulgaris* L., *Arabidopsis thaliana*, *Solanum tuberosum*, and *Spirodela polyrhiza*. Specifically, 7, 6, 5, and 3 *NRAMP* family genes have been identified from these respective species. Notably, the products *PvNRAMP1-7* have been found to possess transporters involved in metal homeostasis in *Phaseolus vulgaris* L. (Ishida et al., 2018). The induction of *StNRAMPs* expression appears to be unique to HMs, suggesting a potential involvement in the transportation and uptake of HMs such as (Cu, Cd, Zn, nickel (Ni), and Pb (Tian et al., 2021). The SpNRAMP gene’s expression level was affected by Cd stress, particularly in the context of Fe or Mn shortage (Chen et al., 2021).

The NRAMP proteins were grouped into three subgroups by phylogenetic analysis, which aligns with recent research that made a similar division among seven *P. vulgaris* NRAMP proteins (Ishida et al., 2018). A comprehensie set of eleven NRAMP members (*PtNRAMP1–11*) were discovered in *Populus trichocarpa*, a widely used woody model plant. These members were further categorized into three groups through phylogenetic analysis (Ma et al., 2023). Additional investigation of the *PtNRAMP* gene structure revealed that members of closely related subgroups typically exhibited comparable exon-intron arrangements, suggesting a shared evolutionary lineage. Additionally, it was shown that the *PtNRAMP* genes in the various subgroups had distinct exon-intron architectures. These modest variations might significantly impact the evolutionary process of these genes. Hence, there is speculation on the involvement of distinct gene architectures in disparate tasks (Ma et al., 2023). The structure of genes has undergone changes during evolutionary processes, leading to an increase in the number of exons/introns. Generally, genes with fewer introns go through the editing process and leave the nucleus sooner (Hashemipetroudi et al., 2023; Yaghobi and Heidari, 2023). Based on this, the different gene structures may affect the expression pattern of *NRAMP* genes.

There was an unequal distribution of the 11 *PtNRAMP* genes over the six chromosomes. Only one *PtNRAMP* gene was found on chromosomes 1, 6, and 16, two on chromosome 7, and three on chromosomes 2 and 5. *PtNRAMP5*, *PtNRAMP6*, and *PtNRAMP7* may have been tandemly duplicated during evolution, as shown by their clustering on chromosome 5 (Ma et al., 2023). The distribution of the five *StNRAMP* genes is observed throughout five chromosomes, specifically Chr00, Chr02, Chr04, Chr09, and Chr1(Tian et al., 2021). Cis-acting elements distinctly regulate gene transcription and expression in plants (Chen et al., 2021). The promoter regions of *NRAMPs* were shown to possess several stress response and hormone response components. Since numerous stressors can affect a gene’s expression, *NRAMP* genes may be involved (Chen et al., 2021). Examining cis-acting elements has indicated that *StNRAMP2* possesses more elements responsive to MYB transcription factors. The sensitivity of *StNRAMP1* to the five HMs in the stem tissue was negligible, resulting in no statistically significant variation in relative expression. Furthermore, it has been noted that the gene’s 2000 bp upstream region exhibits supplementary elements that are receptive to phytohormones, including methyl jasmonate, gibberellin, salicylic acid, and auxin. Consequently, it is plausible that this gene may have played a role in plant growth and development throughout evolution (Tian et al., 2021). According to studies, many phytohormones, including JA, ABA, and SA, have been linked to the plant’s response to various metal stresses (Ma et al., 2023). Additionally, our investigation revealed the presence of several cis-elements linked with HMs in the promoters of the *KoNRAMP* gene. These cis-elements might play a role in the transportation and uptake of metallic substances. The findings of this study align with prior research, demonstrating the presence of multiple cis-acting elements associated with hormone responsiveness within the putative promoter regions of *Spirodela polyrhiza* (Chen et al., 2021), *Solanum tuberosum* (Tian et al., 2021), *Populus trichocarpa* (Ma et al., 2023), *Phaseolus vulgaris* L. (Ishida et al., 2018), and *Arachis hypogaea* L. *NRAMP* genes (Tan et al., 2023).

As several earlier experiments have demonstrated, the entire genome, segmental, tandem, and gene duplication events are necessary for the growth and evolution of gene families (Vision et al., 2000; Grassi et al., 2008; Chen et al., 2021). According to the study conducted by Force et al., 1999 it was found that the likelihood of tandem duplicate events was greater when the complementarity level was higher than segmental duplicates. Furthermore, the duplication events involving tandem and segmental duplications were identified as the primary types of duplication (Kong et al., 2007). According to earlier findings, *Theobroma cacao* exhibited the existence of a single tandem duplicated pair and one pair of segmental duplications (Ullah et al., 2018). *Glycine max* L. showed six duplicated blocks (Qin et al., 2017), while *Oryza sativa* L. demonstrated one syntenic block with the paralogous pair (Wang et al., 2019). Additionally, *A. thaliana* showcased two pairs of segmental duplications (Segond et al., 2009). Nevertheless, the results indicated the absence of tandem duplication in *Kandelia obovata*. Previous investigations have shown similar findings; no tandem duplication was found in *Arachis hypogaea* L. (Tan et al., 2023), and *S. polyrhiza* (Chen et al., 2021). Thus, the number of *KoNRAMP* genes in *Kandelia obovata* may be related to whole-genome duplication during allopolyploid speciation.

In order to get an additional understanding of the transcript levels, we conducted a quantitative real-time polymerase chain reaction (qRT-PCR) to assess the expression of *KoNRAMP* genes in response to various Cu stress conditions. Our findings show that the expression levels of *KoNRAMPs* varied significantly in response to five distinct CuCl_2_ treatments, exhibiting upregulation and downregulation. In leaves, the transcript abundance of *KoNRAMP1*, *KoNRAMP4*, and *KoNRAMP5* decreased in response to CuCl_2_ treatment. The experimental findings revealed that the expression of all *SpNRAMP* gene families was downregulated in response to 50 µM Cd treatments (Chen et al., 2021). The expression patterns of *StNRAMP* exhibited significant variations across different tissues in response to various HM treatments. The expression pattern of *StNRAMP2* is comparable under stress induced by Pb and Cu, as evidenced by the peak in relative expression seen at 24 hours (Tian et al., 2021). As Tian et al. (2021) reported, these findings supported the notion that *NRAMPs* were responsible for the intake and transport of HMs. The results of our study indicate that the expression levels of the *KoNRAMP2* and *KoNRAMP3* genes were significantly increased in response to Cu concentrations of 100 and 200 mg/L. The results of this study were in agreement with prior research. Specifically, the expression levels of *CsNRAMP1*, *CsNRAMP2*, *CsNRAMP9*, and *CsNRAMP10* were shown to be up-regulated in leaves. Additionally, *CsNRAMP2* exhibited a highly responsive reaction to Pb treatment, indicating its heightened sensitivity (Li et al., 2021). Under the stress conditions of Cd and Fe, most *PtNRAMPs* genes exhibited up-regulation in both leaves and roots, except for *PtNRAMP3* and *PtNRAMP8*. In addition, it was shown that *PtNRAMP1*, *PtNRAMP3*, *PtNRAMP6*, *PtNRAMP7*, and *PtNRAMP10* exhibited up-regulation in both leaves and roots in response to Zn and Mn stress, as reported in the study (Ma et al., 2023). The findings of this study align with other research, suggesting that *NRAMPs* play a crucial role in the absorption and transportation of HMs (Tian et al., 2021). In order to enhance the validation of the functionality of the *KoNRAMP* genes, it is important to conduct a series of experiments involving gene overexpression and knockdown.

## 5 Conclusions

**Figure 10:**
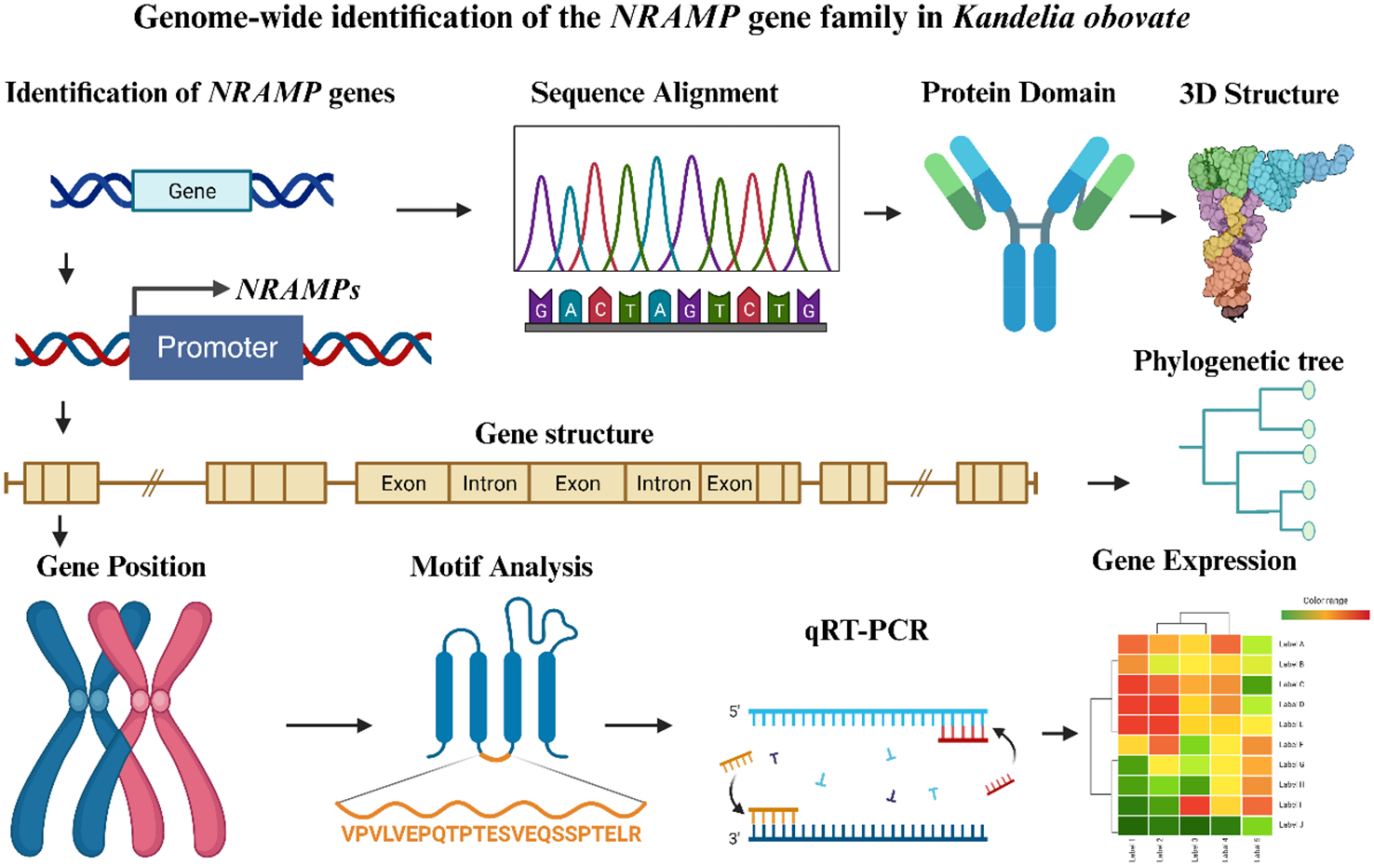
An overview of the thorough investigation of the NRAMP gene family in *Kandelia obovata*. The figure was created using the BioRender (https://www.biorender.com/) tool, which was modified from Sajjad et al. (2023). Different colors were used to illustrate the sketches from the various analyses. The arrow symbol represents the several analyses conducted to comprehend the characterization of the *KoNRMAP* genes.

This work identified five *KoNRAMP* genes by doing a thorough genome-wide analysis of the NRAMP gene family in *Kandelia obovata*. On the five chromosomes of *Kandelia obovata*, these *KoNRAMP* genes were located. The expression patterns of five *KoNRAMP* genes were validated using qRT-PCR. Comparing the Cu400 condition to the Cu0 condition showed that the expression levels of all five genes were considerably down-regulated. Based on phylogenetic analysis, the *KoNRAMP* genes were divided into three subgroups (subgroup I, II, SLC). To better understand the evolution of the NRAMP gene family in the *Kandelia obovata* genome, various analyses were conducted, including domain and 3D structural variation, gene structure, phylogenetic and synteny, chromosomal distributions, motif analysis, subcellular localization, cis-regulatory elements, and expression profiling against CuCl_2_ stress treatments (**Figure 10**). Multiple promoter investigations have revealed the presence of many stress and hormone response elements, which play a crucial role in the response to Cu and other metal-induced stress. In general, this research offers significant insights for future functional investigations about the biological functions of *NRAMP* genes in *Kandelia obovata*.

## Data availability statement

The original contributions presented in the study are included in the article/supplementary material. Further inquiries can be directed to the corresponding author.

## Author contributions

QH: Conceptualization, Writing – original draft, Writing – review & editing. TY: Formal analysis, Software, Methodology, Writing – review & editing. CS: Funding acquisition, Supervision, Writing – review & editing. SL: Formal analysis, Methodology, Writing – review & editing. AK: Writing – review & editing. JN: Writing – review & editing. A-ZM: Writing – review & editing. ME: Writing – review & editing.
